# Effect of Turning Frequency on the Survival of Fecal Indicator Microorganisms during Aerobic Composting of Fecal Sludge with Sawdust

**DOI:** 10.3390/ijerph20032668

**Published:** 2023-02-02

**Authors:** Musa Manga, Chimdi Muoghalu, Miller A. Camargo-Valero, Barbara E. Evans

**Affiliations:** 1 The Water Institute at UNC, Department of Environmental Sciences and Engineering, Gillings School of Global Public Health, University of North Carolina at Chapel Hill, 166 Rosenau Hall, 135 Dauer Drive, Chapel Hill, NC 27599-7431, USA; 2 BioResource Systems Research Group, School of Civil Engineering, University of Leeds, Leeds LS2 9JT, UK; 3 Department of Construction Economics and Management, College of Engineering, Design, Art and Technology (CEDAT), Makerere University, Kampala P.O. Box 7062, Uganda; 4 Departamento de Ingeniería Química, Universidad Nacional de Colombia, Campus La Nubia, Manizales 170003, Colombia

**Keywords:** fecal sludge treatment, fecal pathogens, viable helminth eggs, viable *Ascaris* eggs, *Salmonella* spp., *E. coli*, *Enterococci* spp., nutrient recovery

## Abstract

The study investigated the effect of turning frequency on survival of fecal indicator pathogens (*E. coli*, *Enterococcus* spp., *Salmonella* spp. and helminth eggs) during fecal sludge (FS) co-composting with sawdust. Dewatered FS was mixed with sawdust and composted on a pilot scale using different turning frequencies—i.e., 3 days (3TF), 7 days (7TF), and 14 days (14TF). Composting piles were monitored weekly for survival of fecal indicator microorganisms and evolution of selected physical and chemical characteristics for 14 weeks. Our results show that turning frequency has a statistically significant (*p* < 0.05) effect on pathogen inactivation in FS compost. The 3TF piles exhibited shorter pathogen inactivation periods (8 weeks) than 7TF and 14TF piles (10 weeks). Temperature-time was found to be the major factor responsible for the survival of pathogens in FS composting piles, followed by indigenous microbial activities and toxic by-products (monitored as NH_4_^+^-N). Our study findings suggest that even at low composting temperatures, the high turning frequency can enhance pathogen inactivation. This is a significant finding for composting activities in some rural areas where suitable organic solid waste for co-composting with FS to attain the recommended high thermophilic conditions could be greatly lacking.

## 1. Introduction

Composting which involves the decomposition of organic matter provides a simple and cost-effective alternative treatment method for organic waste and fecal sludge, especially in the low-resource settlings. It encourages a sustainable and circular economy as it allows for the conversion of organic waste materials into a value-added product (organic fertilizer) [1,2]. The success of the composting process is usually related to the quality of the final product (compost), especially its stability and safety. The quality of compost produced is usually influenced by the composting conditions of which the turning frequency is one of the most important [3]. The turning frequency influences the performance of the composting process as it affects oxygen supply and aeration conditions and in turn the biodegradation rate [4]. Optimizing the turning frequency during composting is very important especially in low-resource settings where a large proportion of the composting facilities are manually operated and adapting the turning frequency to the stage of the process is the most implemented aeration strategy [5]. To this end, several studies have been conducted on the composting of various organic wastes so as to examine the effect of aeration rate and turning frequency on the composting process and pathogen inactivation; however, their results have been contradictory [6,7,8]. In a study by Savage et al. [8], it was observed that high turning frequency of 20 times per month during the composting of swine waste and straw (5% wt/wt) enhanced pathogen inactivation by raising composting temperatures to the thermophilic range of 60 °C within 72 h of setting up the composting stacks. All the pathogens (*Salmonella*, fecal streptococci, and fecal coliforms) were inactivated within 14 days of achieving thermophilic conditions. Similarly, Vinerras et al. [9] found high turning frequency to have enhanced pathogen inactivation during thermal composting of fecal matter. Scott [10] also demonstrated that high turning frequency of two turns every week raised temperatures to 60 °C during composting of night soils and other wastes, and thus enhanced inactivation of protozoa and helminth eggs to non-detectable levels within 21 days of setting up composting piles. Rynk et al. [11] reported that periodic turning of the composting heaps ensures restoration of the porosity and structure of the composting feedstock/material, which accelerates pathogen inactivation and the composting process/biodegradation. The above-reviewed studies have shown that turning enhances pathogen inactivation as well as the quality of the compost.

In contrast to the aforementioned studies, some of the most recent studies have demonstrated that turning of composting piles has no effect on pathogen inactivation [12,13]. For instance, Kone et al. [13] observed that the turning frequencies of 3 days and 10 days had no effect on the inactivation efficiency of helminth eggs and attainment of the specified temperature-time criteria for pathogenic inactivation during composting. This finding contradicts the previous studies. Moreover, most of the above-mentioned investigations on turning frequency have been conducted using animal manure. Literature on the effects of turning frequency on the composting process of fecal sludge (FS) which is also a readily available and high-quality feedstock (with high nutrient content) is rather scant. Yet, the optimum turning frequency mainly varies with the characteristics of the material being composted and the methods of preparation. In the same vein, FS contains extremely high pathogen concentrations [12,14,15,16] and the effectiveness of turning frequency on pathogen inactivation during FS composting is still unclear due to conflicting information in the scientific literature. In view of these considerations, this study investigated the effect of different turning frequencies, i.e., 3 days turning frequency (3TF), 7 days turning frequency (7TF), and 14 days turning frequency (14TF) on pathogen (*E. coli*, *Enterococcus* spp., *Salmonella* spp. and helminth eggs) inactivation during FS composting with sawdust. This study findings will provide policy guidelines for operating decentralized FS composting facilities in low-resource settlings, especially in the rural areas where communities are interested in reusing human waste in agriculture.

## 2. Materials and Methods

### 2.1. Composting Plant

The study was conducted at a pilot scale composting facility constructed at National water and sewerage corporation (NWSC) fecal sludge treatment facility at Lubigi, Kampala, Uganda. Kampala is the largest and capital city of Uganda with a population of about 1.8 million people [12]. It is located on the northern shores of Lake Victoria at an altitude of 1223 m above mean sea level (geographic coordinates 0°18′ 58.18′′ N latitude, 32°34′ 55′′ E longitude) [12]. Details on the design and construction of the composting facility are presented in our previous work [17,18].

### 2.2. Compost Set Up and Monitoring

In this study, fecal sludge and sawdust were co-composted. Fecal sludge was collected from ventilated improved pit (VIP) latrines and septic tanks in informal settlements surrounding Lubigi FSTP—i.e., Makerere, Kikoni, and Bwaise. The VIP latrine FS and septage were thoroughly mixed in a ratio of 1:2 by volume (VIP latrine sludge: septage) and then dewatered on sludge drying beds to about 20–35% total solids content prior to composting [14]. Sawdust which was used as the bulking agent in this study was obtained from Bwaise sawmill located about 500 m from the project site and prior to composting, the sawdust was sorted to remove any inorganics.

Dewatered FS (27–35% total solids content) was mixed thoroughly with sawdust (1:2 *v/v*; dewatered sludge: sawdust) to produce six composting static piles of about 3 m^3^ each. The composting piles were aerated by manual turning, with 3-, 7- and 14-days turning frequencies designated as 3TF, 7TF and 14TF, respectively. Each of the turning frequencies were used for operating two composting piles. Daily temperature measurements were carried out for the composting piles at the: top (750 mm from the pile base), middle (400 mm from pile base) and bottom (200 mm from pile base), using a TFA (D-Wertheim, Model 19.2008) stainless steel body compost thermometer. For effective process control, the moisture content of the piles was maintained at 50–60%, air temperature at 19–26 °C and relative humidity at 69–80% [18]. The composting piles were monitored for a period of 15 weeks.

### 2.3. Compost Sampling and Analysis

#### 2.3.1. Sampling Method

Dewatered FS was obtained from the sludge drying bed using the sampling procedure documented in our previous study [17]. During the composting cycle, compost samples of about 400 g each were collected from the top (750 mm from the pile base), middle (400 mm from pile base), and bottom (200 mm from pile base) as well as the outer and inner sections of each composting pile after which they were mixed homogeneously to form a composite sample. Using a quarter sampling method, the homogeneously mixed compost sample was spread on a clean flat surface in a circular pattern and divided into four quadrants after which samples were taken from the center of each the four quadrants and thoroughly mixed again. Thereafter, approximately 500 g of the sample was collected and transported to the laboratory for analysis. The samples were collected at day 0 and subsequently weekly from the composting piles until the end of the composting period. Immediately after collection, the samples were taken to the laboratory and were analyzed within 4 h to minimize changes in microbial population. The analyses were carried out at Bugolobi NWSC central laboratory and the Microbiology laboratory in the Faculty of Veterinary Medicine at Makerere University in Kampala, Uganda.

#### 2.3.2. Physicochemical Analysis

Moisture content (%) was computed by gravimetric method using the difference between the sample’s initial and final weights after oven drying at 105 °C for 24 h following the procedure provided by Okalebo et al. [19]. Ammonium-nitrogen (NH_4_^+^-N) was determined by spectrophotometric method by extracting with 0.5 M K_2_SO_4_ in 1:10 (*w/v*) from fresh compost samples according to procedures reported in the literature [19,20]. The microbial respiratory activity in compost samples was measured based on CO_2_–C mineralization conducted in closed bottles based on the methods documented by Ohlinger [21] for soil respiration techniques, but with some modifications made to techniques based on similar soil respiration procedures reported in the literature [22,23]. Compost was placed in a closed bottle and the CO_2_ which evolved was trapped in an alkaline solution (KOH). The absorbed CO_2_ concentration was then determined by titration with hydrochloric acid solution (0.5 M). The CO_2_–C production rate was assessed and expressed as mg CO_2_–C per mass of organic matter (as Volatile Solids–VS) per day [23].

#### 2.3.3. Microbial Analysis

Sawdust, dewatered FS, and compost samples were analyzed for helminth eggs (*Ascaris* eggs), *Salmonella* spp., and fecal indicators (*Enterococcus* spp. and *E. coli*). The choice of these fecal pathogen indicators stems from the fact that they have been widely documented to be relevant in the assessment of microbial and public health risks associated with the re-use of various waste streams such as animal manure, FS, sewage sludge, etc. as organic fertilizers [22,23,24,25]. Culturable pathogen indicators were determined using the dilution plate count method and the resulting counts were expressed as Log_10_ CFU per g of sample dry weight (dwt). In preparing the samples for analysis of fecal pathogen indicators, a well-mixed sample of about 25 g and 225 mL of peptone H_2_O was homogenized in a sterile stomach bag using a stomacher/pulsifier at 12,000× *g* for 2 min [26]. Thereafter, ten–fold (10^−1^) serial dilutions of the homogenate were prepared and then used for the identification and enumeration of different fecal pathogen indicators. *Salmonella* spp., *E. coli* and *Enterococcus* spp. were analyzed according to standard methods for the examination of water and wastewater [27] using Xylose Lysine Deoxycholate Agar, sterile *E. coli*—Coliforms Chromogenic Agar and sterile Bile Esculin Azide Agar, respectively. Detailed procedures used in this study for analyzing *Salmonella* spp., *E. coli* and *Enterococcus* spp. are presented in our previous work [24,28]. Viable *Ascaris* eggs concentrations were analyzed according to USEPA [29] egg floatation technique. Detailed procedure for viable *Ascaris* eggs analysis used in this study is presented in our previous work [17,18].

#### 2.3.4. Statistical Analysis

Descriptive statistics (mean values ± standard error) were used in reporting the results of the laboratory analysis. Data were analyzed using non-parametric Friedman test. The significance of differences amongst the mean values was tested with 95% confidence interval. Spearman’s rho test was used for examining whether and to what extent the relationship between parameters exists, using a 95% confidence interval. Standard multiple regression analysis was conducted according to Pallant [30], to determine the most important factors responsible for viable *Ascaris* eggs inactivation during composting. Statistical analysis was carried out using IBM SPSS version 21.0 for Windows.

## 3. Results and Discussion

### 3.1. Characterisation of Feedstock

The characteristics of dewatered FS and sawdust which were used as feedstock in the composting process are summarized in Table S1. *Salmonella* ssp., *E. coli*, *Ascaris* eggs and *Enterococci* spp. were not detected in sawdust which was used as the bulking agent for the composting process indicating that it is free from fecal contamination. The average *Salmonella* ssp., *Enterococci* spp. and *E. coli* content found in dewatered FS were 7.3, 6.6 and 7.9 log10 CFU/g dwt, respectively, while the viable *Ascaris* eggs count was 37 ± 16 eggs/g dry weight. These results are in line with data reported in the literature [14,30,31]. However, the helminth egg count for the dewatered FS is far greater than the recommended value for materials used in agriculture as per WHO’s guidelines (≤3–8 eggs g^−1^ TS) [32], indicating that it cannot be directly applied in agriculture without proper treatment. Further, the moisture content found in samples of sawdust (31.2% ± 5.9) and dewatered FS (68.7% ± 3.8) compared well with 13.71–64.2% (sawdust) and 70% (dewatered FS) reported in previous studies [30,32].

### 3.2. Changes in Physicochemical Properties

#### 3.2.1. Temperature

**Figure 1:**
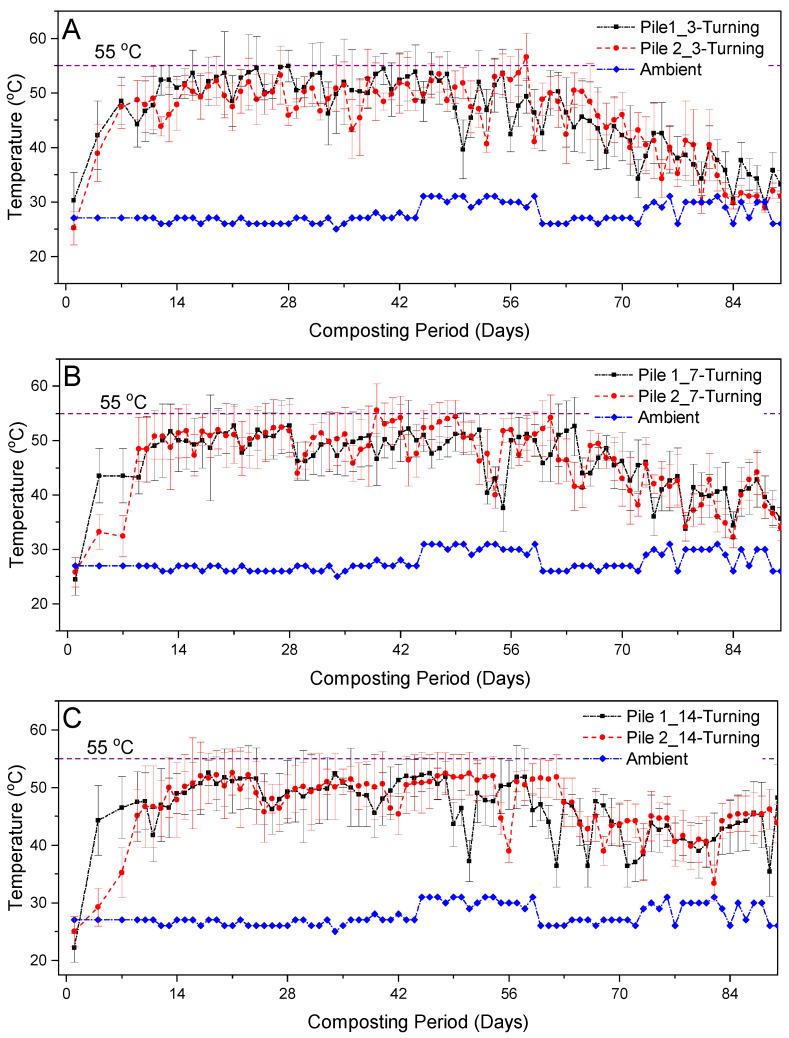
Temperature evolution during composting of Fecal Sludge (FS) and sawdust using (**A**) 3 days turning frequency, (**B**) 7 days turning frequency, (**C**) 14 days turning frequency. In (**A**–**C**) error bars represent the standard deviation of the bottom, center, left side and right-side pile temperatures.

The evolution of average temperatures during the composting of FS with sawdust using three different turning frequencies is shown in Figure 1. It can be noted that all the composting piles failed to attain the average temperatures ≥ 55 °C as their maximum average temperatures were 54 °C, 52.8 °C and 52.1 °C, and these were attained after 27-, 42- and 48-days composting period, respectively. The inability to reach high temperature ranges may have been due to high heat losses to the ambient environment, which can be attributed to the highly porous nature of the composting materials. However, in some sections (middle section) of the 3TF, 7TF and 14TF composting piles, temperatures ≥ 55 °C were attained after 11–19 days composting period with the 3TF composting piles requiring a slightly longer composting period to reach such temperatures (Figure 1). This delayed rise of temperature in 3TF piles could perhaps be explained by the cooling effect caused by significant water and heat loss to the environment in the form of convection and evaporation as a result of excessive turning of the piles. 3TF piles recorded slightly higher average temperatures than 7TF and 14TF piles. This response can be attributed to high turning frequency that may have introduced more oxygen in composting piles for microbial activity or favored the growth and metabolic activities of microbes, which are exothermic. A similar phenomenon was observed by Caceres et al. [33] during the composting of the solid fraction of cattle slurry using different aeration approaches. In addition, during composting, the turning operations caused some interruptions in the temperature profile of all composting piles, with a sudden drop immediately after each turning, followed by a rapid recovery of high temperatures within a 24 h composting period (Figure 1). This depicted the recovery of metabolic activities of microorganisms, which might have been due to the increase in the supply of readily available organic substrate for microbial biomass by incorporation of less degraded material on the outer layers into the inner section of the composting piles. This phenomenon has also been observed by previous researchers [7,34,35]. It can also be noted that temperature changed greatly in the 14TF piles (Figure 1). This significant variations in composting temperatures of 14 TF piles may be attributed to the low turning frequency and thus, uneven conditions may have existed within the composting piles leading to significant variations in the temperature especially after every turning. In this study, we observed that after every turning the composting temperature increased greatly in the 14 TF piles.

#### 3.2.2. pH

**Figure 2:**
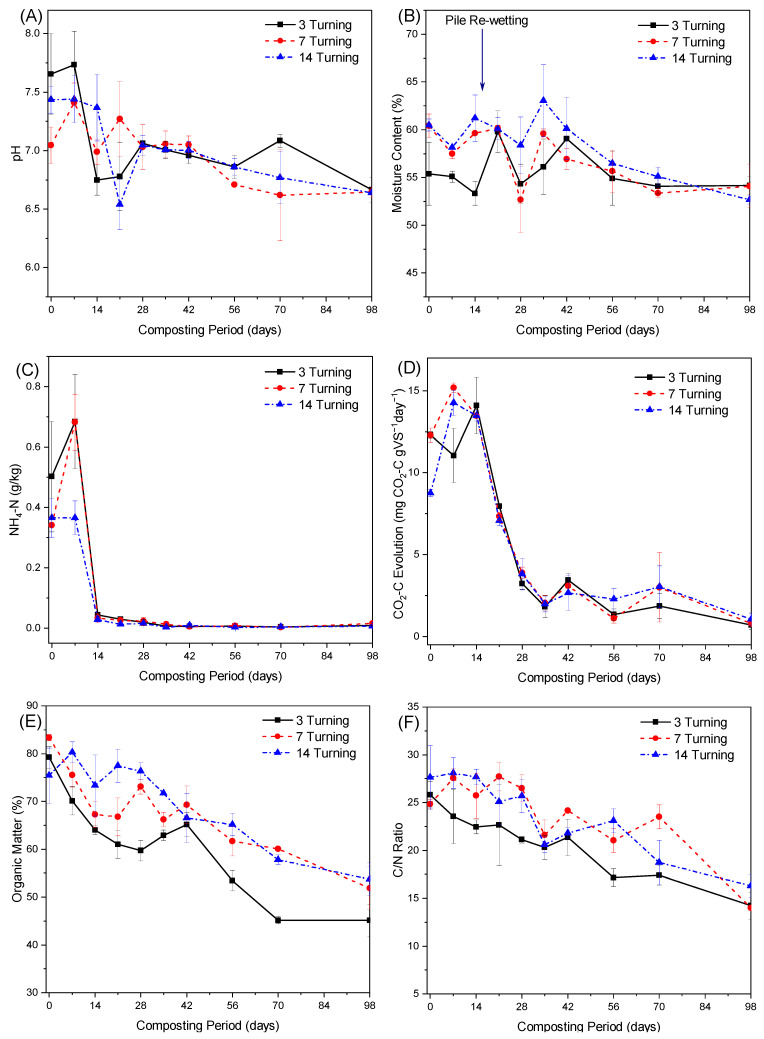
Evolution of (**A**) pH, (**B**) Moisture Content, (**C**) Ammonia nitrogen (NH_4_^+^-N), and (**D**) Carbon dioxide (CO_2_-C) respiration rate, (**E**) Organic matter, and (**F**) C/N ratio during Fecal Sludge (FS) composting with sawdust using 3 days turning frequency—3 Turning, 7 days turning frequency—7 turning, and 14 days turning frequency—14 turning. Error bars represent the standard error of n = 2.

All composting piles exhibited similar pH evolution trend, with the pH values of the 3TF, 7TF and 14TF increasing and then decreasing as they moved from initial values of 7.6, 7.0 and 7.4 to 6.48, 6.58, and 6.72, respectively, at the end of the composting period (Figure 2A). The three turning frequency compost types exhibited increases in the pH values, especially during the thermophilic phase (Figure 2A). This response might be attributed to ammonium formation from NH_3_-N solubilization, and microbial activities during the thermophilic phase. These facts were also confirmed by Spearman’s rho test, which revealed a strong positive relationship between the evolution of pH and NH_4_-N concentration (3TF (*p* = 0.037, R^2^ = 0.446, n = 22), 7TF (*p* = 0.004, R^2^ = 0.595, n = 22), 14TF (*p* = 0.005, R^2^ = 0.572, n = 22)) and significant positive correlation between pH evolution and microbial activities monitored as CO_2_-C evolution (3TF (*p* = 0.018, R^2^ = 0.499, n = 22), 7TF (*p* = 0.0001, R^2^ = 0.741, n = 22) and 14TF (*p* = 0.001, R^2^ = 0.653, n = 22)) during FS composting. Similar findings were found by previous authors [2,33,36]. However, from Figure 2A, it can be seen that 14TF piles exhibited generally stable pH values during the first two weeks of composting while a significant increase was observed in 3TF and 7TF piles. This coincided with the low NH_4_-N concentrations observed in such piles during these composting periods. This phenomenon could perhaps be explained by the low turning frequency, which may have limited the microbial activities responsible for organic matter biodegradation and organic nitrogen mineralization, consequently resulting in the low NH_4_-N formation (Figure 2C) during these composting periods.

#### 3.2.3. Moisture Content

During the composting of 3TF, 7TF, and 14TF piles, the moisture content dropped initially from 55.4%, 60.4%, and 60.5% to 47%, 50% and 51.6%, respectively, by the end of the composting process (Figure 2B), corresponding to moisture loss of 15.2%, 17.7%, and 14.6%, respectively. The decrease in the moisture content for all the piles can be attributed to moisture vaporisation due to the high composting temperatures or high heat generated during the intensive decomposition phase of organic substances. This behavior has also been observed by previous authors [37,38,39]. Furthermore, from Figure 2B, it can be noted that 3TF and 7TF piles exhibited slightly higher moisture content losses than 14TF piles. This may perhaps have been due to the significant moisture losses in the form of water vapor as a result of high turning frequency. Kalamdhad and Kazmi [40] and Margesin et al. [41] similarly found high turning frequency to have resulted into considerably greater moisture losses than the low turning frequency during the composting of organic waste and sewage sludge, respectively. The final moisture content (46.9–51.6%) attained in this investigation compares well with that obtained by Cooperband and Middleton [42] and Michel et al. [43], which were in the range of 40–50% and 42.7–56.4%, respectively. Despite the decrease in the final moisture content, from time to time, all composting piles exhibited an increase in the moisture content especially 14TF piles (un-frequently turned piles) (Figure 2B). This response may perhaps be explained by the organic matter oxidation equation, which may have led to the accumulation of water produced as a by-product of the decomposition process within the composting material. This phenomenon was also observed by earlier authors [44].

#### 3.2.4. Ammonium-Nitrogen (NH_4_^+_^N)

The evolution of ammonium-nitrogen (NH_4_-N) concentration was found to be similar in all the composting piles (Figure 2C). Initially, NH_4_-N concentrations for 3TF, 7TF, and 14TF composting piles of 0.502, 0.341 and 0.365 g/kg increased sharply, reaching peak values of 0.684, 0.682 and 0.366 g/kg on the 7th day, followed by a sudden drop within the second week, and thereafter with a gradual decrease to the stable values of 0.001, 0.007 and 0.007 g/kg, respectively, at the end of the composting process (Figure 2C). The sudden drop can be mainly attributed to significant NH_4_-N losses via higher NH_3_-N volatilization due to high turning frequency, high pH (>7.0, Figure 2A), and the high composting temperature (>40 °C, Figure 1) observed in such piles.

It is important to note from Figure 2C, that 3TF and 7TF piles exhibited a higher NH_4_-N concentration on the 7th day than 14TF, and this was in synchrony with the composting temperature pattern. This may perhaps have been due to the low turning frequency of the 14TF piles, which may have limited microbial activities (responsible for degradation of nitrogen containing compounds) and establishment of conditions (such as high pH and high temperature) that favor the formation of NH_4_-N content within the composting piles. It is also thought that low turning frequency may have led to the formation of anaerobic conditions within the composting piles, and these conditions are associated with low pH values, which limit the formation of NH_4_-N content [45,46].

#### 3.2.5. Carbon Dioxide (CO_2_-C) Respiration Rate

Carbon dioxide (CO_2_-C) respiration rate is usually used as an indicator for evaluating the compost stability and maturity [47]. The respiration rate for 7TF and 14TF piles decreased from 12.3 and 8.8 mg CO_2_-C g VS^−1^day to stable values of 0.46 and 0.69 mg CO_2_-C g VS^−1^day, respectively, by the end of the composting period (Figure 2D). In contrast, the CO_2_-C respiration rate of 3TF piles dropped from 12.3 to 11.1 mg CO_2_-C g VS^−1^day to the stable value of 0.5 mg CO_2_-C g VS^−1^day by the end of the composting period (Figure 2D). It can also be noted from Figure 2D that all the composting piles reached the stable low CO_2_-C respiration rate values at almost the same composting periods implying that the turning frequency had no effect on the compost maturity. This finding is in agreement with that of Michel et al. [43], who similarly found that turning frequency (of 4 days and 28 days) had no significant effect on microbial respiration rate measured as oxygen uptake rates. A more comprehensive discussion on CO_2_–C evolution results observed in the composting piles used in this study is being presented in our work elsewhere [48].

#### 3.2.6. Organic Matter

Figure 2E illustrates the evolution of organic matter (OM) during the composting of FS with sawdust using different turning frequencies. The OM content in all composting piles was somewhat similar in the range of 75.4–84.3% but these significantly differed within piles towards the end of the composting process, falling within the range of 39.7–52.5% (Figure 2E). The final OM (39.7–52.5%) concentrations recorded in this investigation are in line with results obtained by Michel et al. [43] from composting of yard trimmings using different turning frequencies (OM of 38.1–49.9%). The OM content decreased steadily as the composting process progressed; 3TF and 7TF piles exhibited lower OM content than 14TF piles throughout the composting process. The higher OM content of the 14TF piles can be attributed to the low turning frequency and unfavorable conditions in such piles, which may have limited the aerobic biological or microbial activities responsible for the degradation of organic matter within the composting piles.

#### 3.2.7. Carbon to Nitrogen (C/N) Ratio

The carbon to nitrogen (C/N) ratio of all the composting piles declined as the composting process progressed, regardless of the turning frequency; the C/N ratio of 3TF, 7TF, and 14TF piles decreased from initial values of 25.8, 24.8, and 27.6 to 9.3, 9.7, and 11.5, respectively, by the end of the composting process (Figure 2F). Similar behavior has been observed by several previous researchers during the composting of different organic wastes [47,48,49,50,51]. It can also be noted from Figure 2F that all composting piles exhibited slight fluctuations in the C/N ratio values, especially during the thermophilic phase. The decreases in the C/N ratio can be attributed to the decomposition of organic carbon and increase in the concentrations of total nitrogen (TN) while the increases can be largely linked to nitrogen losses via NH_3_-N especially during the thermophilic phase.

### 3.3. Pathogen Inactivation (E. coli, Salmonella spp., Enterococcus spp., Helminth Eggs)

#### 3.3.1. *Escherichia coli* (*E. coli*)

**Figure 3:**
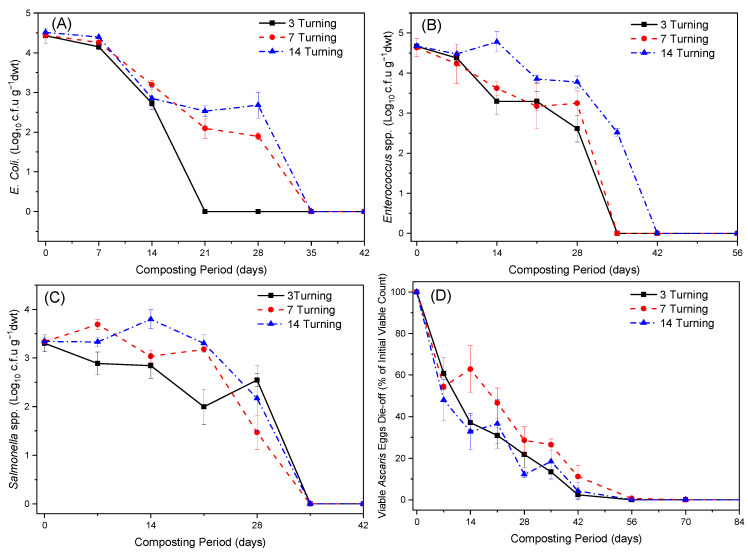
Changes in (**A**) *E. coli* population, (**B**) *Enterococcus* spp. population, (**C**) *Salmonella* spp. population and (**D**) Viable helminth eggs (*Ascaris* eggs) during the composting of FS and sawdust using 3 days turning frequency—3 Turning, 7 days turning frequency—7 Turning, and 14 days turning frequency—14 Turning. Error bars represent the standard error of n = 2.

Owing to the fact that *E. coli* is the most representative member of the fecal coliforms group [52], it is one of the pathogenic microorganisms indicators commonly monitored in biological processes used for treating organic wastes containing fecal related material. All the three turning frequency composting piles initially exhibited a slow decrease in the *E. coli* population. This can be attributed to the low temperatures (<45 °C) observed in these piles during the first week of composting, which could not have brought about significant thermal destruction of these microorganisms (Figure 3A). This clearly shows that temperature is a major factor responsible for the thermal destruction of these pathogens during composting. Similar results were observed by Christensen et al. [53] during the composting of sewage sludge with yard waste/ straw in the open-air windrow, where they found that the low mean composting temperatures of 44.9 °C at the base of the windrows coincided with a very low reduction in the *E. coli* population.

In the present study, the turning frequency proved to have a significant effect on the survival of *E. coli* during FS composting. The 3TF piles attained shorter *E. coli* survival periods of 3 weeks compared to 7TF and 14TF piles with 5 weeks (Figure 3A). This finding might be attributed to the high turning frequency used, which may have promoted even distribution and exposure of these pathogens to the high temperatures within the composting piles. A remarkable drop in *E. coli* viable count can be observed in the 3TF piles at 21 days of composting (Figure 3A). This drop may be attributed to the fact that the thermal death of different microorganisms occurs through various mechanisms. For *E-coli*, its thermal destruction occurs by deformation of the nucleoids while for other microorganisms, thermal destruction occurs by rupture of the cell envelope [54]. The different mechanisms for thermal death imply that the rate of death will be different for the different microorganisms. The study results suggest that high turning frequency promotes multiple cycles of heating, which may be more effective at destroying pathogens at relatively lower temperatures than the low turning frequency that exposes them to a single or few high-temperature cycles. This observation is in agreement with Hess et al. [55]. However, further research is still needed to confirm this observation and also to determine the optimum cycles needed for complete thermal destruction of pathogens at low temperatures. Hutchison et al. [56] reported contradicting results, as they found aerating of piles to have not had a significant effect on the inactivation efficiency of inoculated *E. coli* during the composting of livestock waste and spent bedding in static heaps. They attributed this to the rapid decline in pathogens levels that occurred within the first 2 weeks of composting. The *E.coli* inactivation periods attained in this experimental trial are consistent with those published by previous researchers [57].

Furthermore, although previous studies have reported a rapid reduction in the *E.coli* population to occur during the thermophilic phase where high temperatures (>55 °C) exist in the composting piles [55,58], this behavior was not observed in the present study, especially in the 14TF piles. These piles recorded a retarded decrease in *E. coli* population, where temperatures close to 55 °C were observed in the piles between the 3rd and 4th week of composting. This peculiar behavior is thought to have been due to the uneven distribution of lethal temperatures within the composting piles from the low turning frequency. On the other hand, this surprising response can be attributed to the changing of the nutrient status of the composting feedstock, thereby changing the competition pressure within the composting piles [59]. A similar finding has been registered by Chroni et al. [60] during the composting of source-separated bio-waste in a pilot plant; they found these pathogens to have survived for over 57 days, despite the high temperatures (67 °C) reached by the 25th day. A more surprising result was published by Pourcher et al. [61], who similarly found *E. coli* to have survived in the composting piles of rural sewage sludge with straw, which attained thermophilic conditions for an extended period of 61 days, with temperatures as high as 69.4 °C reached in some pile sections.

#### 3.3.2. *Enterococcus* spp.

The survival of *Enterococcus* spp. pathogens in the three turning frequency compost types was similar, within the first week of composting. However, this differed significantly thereafter as the composting process progressed due to the difference in the composting conditions caused by different turning frequency configurations (Figure 3B). 3TF, 7TF, and 14TF composting piles demonstrated a decline in the initial *Enterococcus* spp. population of approximately 2.06, 1.39, and 0.9 log cfu/g dwt., respectively, within a composting period of 28 days. This was then followed by significant die-off of these microorganisms, with 3TF and 7TF piles reaching undetectable limits on the 35th day of composting, while the 14TF piles required 7 more days to reach undetectable levels.

The 3TF and 7TF compost piles exhibited higher *Enterococcus* spp. inactivation efficiency than the 14TF, which illustrates that the turning frequency had an effect on the survival of such microorganisms during composting (Figure 3B). This may be attributed to the relatively high thermophilic temperatures values reached and sustained for a relatively extended period in these piles as compared to 14TF piles. On the other hand, the high turning frequency may have ensured sufficient exposure of all compost particles to lethal temperatures, since all composting material on the cooler outside areas, which may have contained surviving *Enterococcus* spp. was frequently incorporated into the center of the composting piles for effective pathogen destruction. Vinneras et al. [9] similarly found frequent turning to have increased the inactivation of pathogens during thermal composting of fecal matter while the longer *Enterococcus* spp. inactivation periods exhibited by 14TF piles may have been due to the non-uniform distribution of temperatures throughout the composting piles from low turning frequency. This was also supported by the statistical test results which showed significant differences in the survival of *Enterococcus* spp. (with *p* = 0.014) caused by the three turning frequency configurations. It is important to note that continued monitoring of *Enterococcus* spp. during the maturation phase, showed no regrowth of these micro-organisms during maturation, which confirmed their complete inactivation during composting.

Furthermore, the 7TF and 14TF piles showed a slight increase in the *Enterococcus* spp. content on the 14th and 28th day of composting, respectively. This phenomenon has also been observed by other researchers. Kroggman et al. [62] detected an increase in the fecal streptococci (*Enterococcus* spp.) content of approximately 10-fold during the composting of horse manure. Sesay et al. [63] similarly noticed an increase in the *Enterococcus* spp. (fecal streptococci) content from 1.8 × 10^3^ to 5.5 × 10^4^ during the composting of municipal solid waste in classical piles, after the second turning. Although some composting studies [64] have attributed this increase in *Enterococcus* spp. (fecal streptococci) during composting to cross contamination, this is thought to have not been the case in the present study, given that all the piles were properly separated from each other and extreme hygienic precautions in collection of samples, and proper housekeeping were maintained throughout the composting process. Thus, this increase in *Enterococcus* spp. content can mainly be explained by secondary growth due to the longer turning frequency, which may have favored the existence of favorable or non-aggressive environmental factors such as slight alkalinization (pH in the range of 8–8.5), moisture content and moderate temperature that support the regrowth of these microorganisms within some sections of the composting piles.

#### 3.3.3. *Salmonella* spp.

During the early composting periods, all the composting piles exhibited a very slow decrease in the *Salmonella* spp. population. The 3TF composting piles exhibited a decrease in *Salmonella* spp. content of ca. 1.31 log cfu/g dwt. from day 0 through day 21 (Figure 3C). Surprisingly, this was thereafter followed by a significant increase in *Salmonella* spp. population of ca. 0.56 log CFU/g dwt. during 21 and 28-days composting period; before it dropped appreciably reaching undetectable levels by day 35. In contrast, the 7TF and 14TF piles demonstrated very slow decline in *Salmonella* spp. during day 0 and 21, recording a die-off of only 0.16 and 0.03 log cfu/g dwt., respectively. This was then followed by a pronounced decline reaching undetectable levels by day 35. The slow decrease in the *Salmonella* spp. content can be attributed to the low temperatures recorded in these piles, which were rather low to cause a significant reduction in *Salmonella* spp. content during those composting periods. Surprisingly, 7TF and 14TF piles also exhibited a slight increase in *Salmonella* spp. content especially during the early stages of composting, though they were not statistically significant. This strange behavior may be attributed to the recontamination or redistribution effect of these microorganisms in the composting material during the turning operations. In addition, the statistical test results revealed that there was no significant difference (*p* = 0.150) in the survival of *Salmonella* spp. caused by the treatment. This finding is in agreement with Tam and Tiquia [50] who found the turning frequency to have not had a significant effect on the survival of *Salmonella* spp. during the composting of pig litter in forced-aerated and turned piles.

Continued monitoring of the *Salmonella* spp. showed that they were completely inactivated after 5 weeks of composting and there was no re-growth of these microorganisms during maturation. The period of 5 weeks is relatively longer than those published by other authors, which are usually in the range of 5–25 days [65,66]. For instance, Knoll [67] attained complete *Salmonella serotype Cairo* inactivation after 7 days at temperatures of 50 °C, during the composting of sewage sludge in a ventilated incubator while Ceustermans et al. [65] achieved complete eradication of *Salmonella Senftenverg strain W 775* after 7 days and 7–14 days at temperatures of 50–55 °C and 40–45 °C respectively, during the composting of garden wastes and biowastes in composting bins and tunnels. The longer *Salmonella* spp. inactivation periods observed in the present study can be attributed to the slower heating rate of the composting piles (Figure 1) that may have induced the production of heat-shock proteins, thus increasing heat resistance of these pathogens during composting. Similar behavior has been observed by some researchers [60,68].

Furthermore, the study results suggest that the slightly acidic conditions (Figure 2A) observed in the piles throughout the entire composting process may have equally played an important role in the inactivation of *Salmonella* spp. especially in 7TF and 14TF piles. This was confirmed by Spearman’s rho correlation test that revealed a significant positive relationship between *Salmonella* spp. inactivation efficiency and acid conditions measured by pH evolutions (3TF (*p* = 0.026, R^2^ = 0.47, n = 22), 7TF (*p* = 0.003, R^2^ = 0.60, n = 22) and 14TF (*p* = 0.010, R^2^ = 0.54, n = 22)) during composting. These acidic conditions in the piles may have been as a result of incorporating high proportions of sawdust in the starting feedstock, which is acidic in nature. In agreement with this study experiment trial results, Mohaibes et al. [69] similarly found the thermal inactivation efficiency of microbial contaminants in food wastes and farm slurry to have improved by operating the thermal treatment process at an acidic pH. This implies that formulation of composting feedstock, to create slightly acidic conditions during composting, may ensure destruction of these pathogens and many others, under circumstances when the composting process fails to generate adequate temperatures for thermal destruction. However, more research is required to confirm this finding, and also to determine the optimum mesophilic conditions and acidic pH values at which effective and faster inactivation of these pathogens can be ensured in large scale implementation.

#### 3.3.4. Helminth Eggs (Ascaris Eggs)

In the 3TF, 7TF and 14TF piles, significant reductions of about 39.2%, 45.6%, and 52.1%, respectively, in the helminth eggs were registered during the first week of composting, where mesophilic conditions with temperatures in the range of 25.1–44.9 °C existed within the composting piles. The helminth eggs inactivation rate improved further during the thermophilic phase, with 3TF and 14TF piles attaining 100% helminth eggs inactivation after 56 days composting period. Although 7TF piles exhibited higher thermophilic temperatures than 14TF piles (Figure 3D), they attained longer helminth eggs inactivation periods of about 70 days (Figure 3D). This is surprising, although it clearly demonstrates that pathogen inactivation in 14TF piles was not solely dependent on the temperature-time parameter; other factors such as microbial antagonism and toxic byproducts released as a result of the indigenous microflora activities may have equally played a significant role in the inactivation of these pathogens. This hypothesis was supported by the Spearman’s rho correlation test, which revealed a significant inverse relationship between the survival of viable *Ascaris* eggs and indigenous microflora activities monitored as CO_2_-C evolution (3TF (*p* = 0.0001, R^2^ = 0.90, n = 22), 7TF (*p* = 0.0001, R^2^ = 0.86, n = 22) and 14TF (*p* = 0.0001, R^2^ = 0.80, n = 22)) as well as the toxic byproducts monitored as NH_4_-N evolution (3TF (*p* = 0.0001, R^2^ = 0.77, n = 22), 7TF (*p* = 0.006, R^2^ = 0.56, n = 22) and 14TF (*p* = 0.0001, R^2^ = 0.79, n = 22)) (see Table S2). On the other hand, the shorter helminth eggs inactivation period attained by 14TF piles may have been due to the longer turning frequency, which allows extended exposure of viable *Ascaris* eggs to moderate–high temperatures in the middle sections of the piles for effective pathogen destruction before turning. The viable *Ascaris* eggs inactivation periods attained in this study are consistent with those reported by Nell et al. [57] during the full-scale composting of sewage sludge in windrow systems, which was 8 weeks.

In the study, the statistical test results revealed that there was a significant difference (*p* = 0.008) in the helminth eggs inactivation efficiency caused by the different turning frequency configurations. However, this result contradicts that of Kone et al. [13], who found the turning frequencies of 3–4 days and 10 days to have had no significant effect on the survival of helminth eggs during the composting of similar feedstock in heaps. This discrepancy in the results could have been due to the difference in the turning frequencies used, differences in the size of the composting piles and the difference in the characteristics of the starting feedstock, composting conditions (aeration rate, climatic conditions, and moisture content) and climatic conditions. On the other hand, it could also be explained by the difference in how the selected turning method was effectively used during each turning operation, and the difference in composting duration to which the different turning frequencies were applied to the composting piles. For instance, in the present study the turning frequencies tested were applied to the composting piles throughout the composting process (both thermophilic and maturation phase) whereas, in the study by Kone et al. [13] the tested turning frequencies were only applied to the composting piles during the thermophilic phase, thus resulting in the discrepancies in the results. Yet, both phases play a significant role in the inactivation of pathogens during composting. Moreover, some studies have shown significant pathogen inactivation to occur during the mesophilic phase due to the activities of microflora such as actinomycetes and fungi.

## 4. Implications of the Study Findings

The data collected in this study on the survival of pathogens and temperature evolution indicate that temperature cannot be easily related/correlated with pathogen inactivation irrespective of the turning frequency used during composting. A similar observation has been made by Droffner and Brinton [70] and Manga et al. [24]. These results suggest that the mechanism for pathogen inactivation during composting is complex and does not depend solely on the temperature-time parameter or thermal conditions—although the temperature-time parameter is the major factor. In the same vein, our study findings suggest that pathogen inactivation in composting piles also depends on other factors such as nutrient depletion, generation of toxic by-products, moisture content, acidic conditions, indigenous microbial activities, and antagonistic effects. (See Supplementary Information, Table S2.) However, further research is needed to determine the extent to which each of the aforementioned factors contributes to pathogen inactivation during composting. Future research should determine the optimum operating conditions for each factor so as to ensure effective and faster pathogen inactivation during full-scale implementation.

Pathogenic indicator organisms are usually used in assessing compost sanitisation so as to avoid the analysis of all types of pathogens during composting [71]. In line with this, the results of this study suggest that *Ascaris* eggs and *Enterococcus* spp. have greater capacity to resist die-off during composting as they exhibited longer survival periods than *E. coli* and *Salmonella* spp. Similar results have been reported by other researchers [53,57]. Viable *Ascaris* eggs survived 7 days longer than *Enterococcus* spp. in some piles, which implies that viable *Ascaris* are more suitable pathogenic indicators for monitoring the survival of pathogens in the composting process especially in rural communities. However, in circumstances where they are not present in the starting feedstock, then the *Enterococcus* spp. can be used instead.

Results of previous studies suggest that for complete die-off of pathogens during composting, composts in windrows or piles should be subjected to temperatures ≥ 55 °C [13,62]. However, the findings of this study suggest that even at lower composting temperatures such as ≥50 °C, the high turning frequency can enhance pathogen inactivation. Therefore, for effective pathogen inactivation during composting, frequent turning of the composting piles is highly recommended such that the surviving pathogens in the non-lethal sections of the composting piles (e.g., outer layers) can be repeatedly transferred to the core sections with high temperatures or lethal conditions for effective destruction of pathogens. This finding has remarkable significance in FS composting since in some rural communities where suitable organic solid waste for co-composting with FS to attain high thermophilic conditions could be lacking, the composting process can be operated at low temperatures but with high turning frequency for effective pathogen inactivation. However, it is important to note that high turning frequency is associated with significant nutrient losses especially nitrogen losses in form of ammonia, thus affecting the nutrient quality of the final product. In the same vein, high turning frequency leads to significant organic matter (OM) and total organic carbon (TOC) losses as there is increased air supply which stimulates the rapid degradation of organic matter as well as bio-oxidation of TOC to carbon-dioxide during composting. Thus, considerations should be given to the choice of turning frequency such that it is not too high nor too low so as to ensure that the compost produced has good quality.

## 5. Conclusions

This study investigated the fate of fecal pathogen indicators during FS composting under different turning frequencies. The following conclusions are drawn from the study’s findings:

- Turning frequency had a significant effect on the survival of *E. coli*, *Enterococcus* spp., and helminth eggs (*p <* 0.05), except for *Salmonella* spp. (*p* = 0.150). This study suggests that high turning frequency promotes multiple cycles of heating, which may be more effective at destroying pathogens at relatively lower temperatures than the low turning frequency that exposes them to a single or few high-temperature cycles.
- Pathogen inactivation is not solely dependent on the temperature-time parameter, but also on other factors such as microbial antagonism (indigenous microbial activities) and production of toxic byproducts (monitored as NH_4_-N).
- Notable additive interactive effects between the physicochemical factors and the different pathogenic indicators were observed. The right combination of multiple physicochemical factors can jointly abate the pathogens.
- *Ascaris* eggs and *Enterococcus* spp. showed longer survival periods than *E. coli* and *Salmonella* spp. indicating that they have higher capability to resist die-off during composting. Hence, they can be used as pathogenic indicators for monitoring the survival of pathogens in the composting process especially in rural communities.
- Regardless of the different turning frequency used, all the composting piles attained lethal conditions suggested for effective pathogen inactivation during composting. 3TF and 7TF exhibited shorter pathogen inactivation periods of 8 weeks than 14TF piles which required 10 weeks. These results suggest that FS compost had been thoroughly sanitized, and could be used for unrestricted agriculture without public health risks.

## Data Availability

Data available as supplementary information: “Data supporting this study are included within the article and/or supporting materials”.

## Supplementary Materials

The following are available online at https://www.mdpi.com/article/10.3390/ijerph20032668/s1, Table S1: Characteristics of feedstock used. Table S2: Spearman’s rho correlation test result between the survival of viable helminth eggs (*Ascaris* eggs) and other mechanisms responsible for pathogen die-off during FS co-composting with sawdust using different turning frequencies.
