# Supplementary material for: Effect of Turning Frequency on the Survival of Fecal Indicator Microorganisms during Aerobic Composting of Fecal Sludge with Sawdust
Source: Int J Environ Res Public Health. 2023 Feb 2;20(3):2668. doi: 10.3390/ijerph20032668 (PMC9915456; doi:10.3390/ijerph20032668)
Supplement: Supplementary file 1 [file ijerph-20-02668-s001.zip › Manga et al _ ijerph-2133685-supplementary Material.pdf]

Effect of turning frequency on the survival of faecal indicator microorganisms during aerobic composting of faecal sludge with sawdust

Musa Manga [1,2,3,*], Chimdi Muoghalu [1], Miller A. Camargo-Valero [2,4] and Barbara E. Evans [2]

[1]The Water Institute at UNC, Department of Environmental Sciences and Engineering, Gillings School of Global Public Health, University of North Carolina at Chapel Hill, 166 Rosenau Hall, 135 Dauer Drive, Chap-el Hill, NC 27599-7431, USA

[2]BioResource Systems Research Group, School of Civil Engineering, University of Leeds, Leeds LS2 9JT, UK.

[3]Department of Construction Economics and Management, College of Engineering, Design, Art and Technology (CEDAT), Makerere University, P.O. Box 7062, Kampala, Uganda. Mobile: +256-702-965158

[4]Departamento de Ingeniería Química, Universidad Nacional de Colombia, Campus La Nubia, Manizales, Colombia

[*]*Corresponding email: mmanga@email.unc.edu*

Table S1: Characteristics of Feedstock used‡

Parameter	*Dewatered FS*	*Sawdust*
Moisture (%)	68.71 ± 3.80	31.20 ± 5.94
Ammonium-N* (mg/kg)	0.53 ± 0.39	0.00 ± 0.01
Escherichia coli (log_{10}/cfu/g dwt)	6.6 ± 0.1	ND
Salmonella spp. (log_{10}/cfu/g dwt)	7.3 ± 0.0	ND
Enterococci spp. (log_{10}/cfu/g dwt)	7.9 ± 0.0	ND
Helminth (Viable *Ascaris* Eggs) (eggs/ g)	37 ± 16	ND

‡Mean ± Standard deviation (SD) of triplicates; * dry base; ND = no detectable

Table S2: Spearman's rho correlation test results between the survival of viable helminth eggs (*Ascaris* eggs) and
other mechanisms responsible for pathogen die-off during FS co-composting with sawdust using different
turning frequencies

**Parameter Evolution**	**3TF, n=22**		**7TF, n=22**		**14TF, n=22**	
	P	**r**	***P***	**r**	***P***	**r**
CO_2-C (mg CO_2-C g VS^{-1}day)	0.0001	-0.897	0.0001	-0.864	0.0001	-0.798
NH_4-N (g/kg)	0.0001	-0.770	0.006	-0.563	0.0001	-0.794
pH	0.001	-0.672	0.014	-0.515	0.003	-0.595
C/N	0.0001	-0.702	0.009	-0.541	0.003	-0.610
Organic matter (%)	0.0001	-0.809	0.0001	-0.771	0.002	-0.613
Moisture Content (%)	0.461*	0.166	0.0001	0.729	0.001	0.639

* Not Significant
